# Doppler ultrasound scoring to predict chemotherapeutic response in advanced breast cancer

**DOI:** 10.1186/1477-7819-5-99

**Published:** 2007-08-28

**Authors:** Anand Kumar, Seema Singh, Satyajit Pradhan, Ram C Shukla, Mumtaz A Ansari, Tej B Singh, Rohit Shyam, Saroj Gupta

**Affiliations:** 1 Department of Surgery, Institute of Medical Sciences, Banaras Hindu University, Varanasi 221005, India; 2 Radiotherapy & Radiation Medicine, Institute of Medical Sciences, Banaras Hindu University, Varanasi 221005, India; 3 Radiodiagnosis & Imaging, Institute of Medical Sciences, Banaras Hindu University, Varanasi 221005, India; 4 Division of Biostatistics, Institute of Medical Sciences, Banaras Hindu University, Varanasi 221005, India; 5 Department of Pathology, Institute of Medical Sciences, Banaras Hindu University, Varanasi 221005, India

## Abstract

**Background:**

Doppler ultrasonography (US) is increasingly being utilized as an imaging modality in breast cancer. It is used to study the vascular characteristics of the tumor. Neoadjuvant chemotherapy is the standard modality of treatment in locally advanced breast cancer. Histological examination remains the gold standard to assess the chemotherapy response. However, based on the color Doppler findings, a new scoring system that could predict histological response following chemotherapy is proposed.

**Methods:**

Fifty cases of locally advanced infiltrating duct carcinoma of the breast were studied. The mean age of the patients was 44.5 years. All patients underwent clinical, Doppler and histopathological assessment followed by three cycles of CAF (Cyclophosphamide, Adriamycin and 5-Fluorouracil) chemotherapy, repeat clinical and Doppler examination and surgery. The resected specimens were examined histopathologically and histological response was correlated with Doppler findings. The Doppler characteristics of the tumor were graded as 1–4 for <25%, 25–50%, >50% and complete disappearance of flow signals respectively. A cumulative score was calculated and compared with histopathological response. Results were analyzed using Chi square test, sensitivity, specificity, positive and negative predictive values.

**Results:**

The maximum Doppler score according to the proposed scoring system was twelve and minimum three. Higher scores corresponded with a more favorable histopathological response. Twenty four patients had complete response to chemotherapy. Sixteen of these 24 patients (66.7%) had a cumulative Doppler score more than nine. The sensitivity of cumulative score >5 was 91.7% and specificity was 38.5%. The area under the ROC curve of the cumulative score >9 was 0.72.

**Conclusion:**

Doppler scoring can be accurately used to objectively predict the response to chemotherapy in patients with locally advanced breast cancer and it correlates well with histopathological response.

## Background

Breast cancer is a leading cause of death in women. In spite of advances in treatment, mortality in this subset of patients is substantial. An advanced local disease in the form of extensive edema of skin, ulceration, large lump and fixity limit the applicability of local treatment alone. Neoadjuvant chemotherapy has been accepted as the primary modality of treatment in locally advanced breast cancer. It offers a definite advantage by down staging the tumor thus allowing less extensive surgery. It also improves survival in chemo-responsive patients and provides better quality of life. Chemo-responsive tumors have a better overall survival than non-responders [1]. It is imperative to evaluate the response to chemotherapy objectively. Imaging modalities like mammography, ultrasound, computed tomography, magnetic resonance imaging and radioisotopes are used for evaluation of breast [2,3]. Ultrasonography (US) is used primarily for diagnostic purposes and for wire localization biopsy of atypical or suspicious lesions. With the technological advances in sonographic imaging, US can now be used as an alternative method for assessment of the tumor including the response to primary systemic treatment [4]. Color Doppler is also used in the assessment of tumor vascularity since it can detect vessels as small as 0.2 mm in diameter [5]. The Doppler Sonographic study of vascularity includes assessment of parameters like number of flow signals, peak flow velocity (Vmax), resistivity index (RI), and pulsatility index (PI). It also permits noninvasive visualization of abnormal vessel architecture in breast tumors referred to as neoangiogenesis. The change in vascularity of the tumor corresponds to the histopathological response and therefore the study of vascularity can be used as an objective complementary tool for assessing response to therapy in locally advanced breast cancer. The purpose of this study was to propose a Doppler scoring system which would provide a quantitative method of assessment and predict the response which would help in monitoring primary chemotherapy in locally advanced breast cancer and prognosis.

## Patients and methods

This prospective clinical study included 50 patients with locally advanced breast cancer who received neo-adjuvant chemotherapy in a single surgical unit at a tertiary level university teaching hospital in North India between January 2001 and January 2005. The Institute postgraduate research board and the departmental research committee have approved the study and the informed written consent of the subjects was recorded individually on the case records. The included patients had T3 or T4 lesions with positive histology. Patients were evaluated clinically prior to chemotherapy and surgery. They received 3 cycles of neoadjuvant chemotherapy (a combination of Cyclophosphamide, Adriamycin and 5-Fluorouracil) at three weekly intervals prior to mastectomy which was performed in all patients. Color Doppler examination of the tumor was done using LOGIQ 400 CL System (GE medical system) with a LA 39, 11 MHz probe and the parameters were recorded. Ultrasound examination was performed by a single experienced sonologist who was blinded to the patients' clinical profile, treatment history, response status and the pre chemotherapy findings. The scan was done in multiple planes to include whole of the breast and axilla. Standardised machine setting were used to optimise sensitivity to low velocity and low volume blood flow. The Doppler scan was done to evaluate intra-tumoral flow signals, **PI** i.e. Peak flow velocity - End diastolic velocity/average velocity, **RI **i.e. Peak systolic velocity - End diastolic velocity/Peak systolic velocity and **Vmax**. The number of flow signals was assessed manually by counting the pixels inside the tumor mass. Tumor response evaluation was done as per the RECIST guidelines [6]. The Doppler findings were graded as 1–4 and the grading was done with reference to the Doppler assessable parameters (Percentage change in RI, PI & Vmax) individually: Grade 1 – Increase/no change/less than 25% reduction (given score-1); Grade 2 – 25–50% reduction (given score-2); Grade 3 – More than 50% reduction but not complete disappearance (given score-3); Grade 4 – Complete disappearance (given score-4). Histopathological response grading was done as: Grade 1 – No chemotherapeutic change; Grade 2 – Minimal chemotherapeutic change (<50%); Grade 3 – Moderate chemotherapeutic change (>50% but <100%); Grade 4 – Complete annihilation of tumor (100% disappearance of tumor). The individual grades of RI, PI & Vmax for a patient were added and the cumulative scores were evaluated at different cut off points, i.e. >5, >7 and >9. The sensitivity, specificity, PPV, NPV were calculated at different Grades, i.e. >1, >2 & >3 of RI, PI & Vmax and compared to histopathology grade 4 which was taken as the gold standard. Chi square test (χ^2^) was also used for analysis of results. ROC curves were obtained for the RI, PI, Vmax scores individually and also for the cumulative score of the Doppler parameters.

## Results

**Figure 1:**
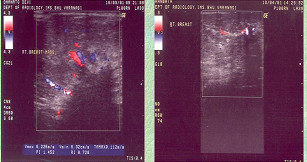
Partial tumor disappearance on color Doppler.

**Figure 2:**
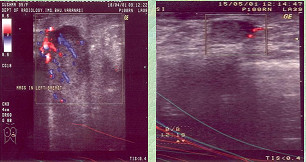
Complete tumor disappearance on color Doppler.

**Figure 3:**
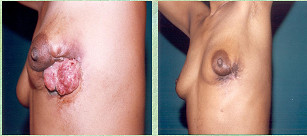
Clinical photograph of the patient.

**Figure 4:**
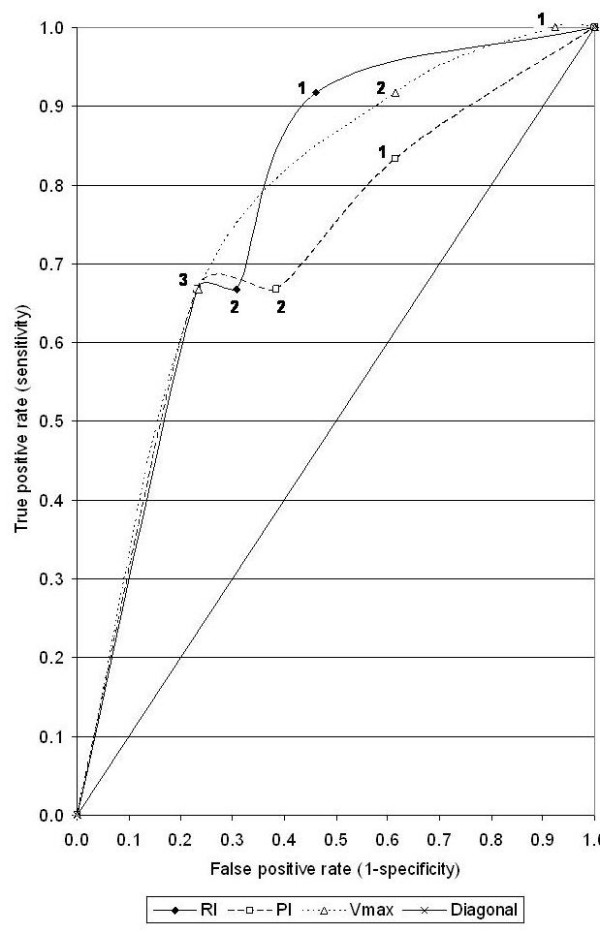
ROC curve for RI, PI and Vmax.

**Figure 5:**
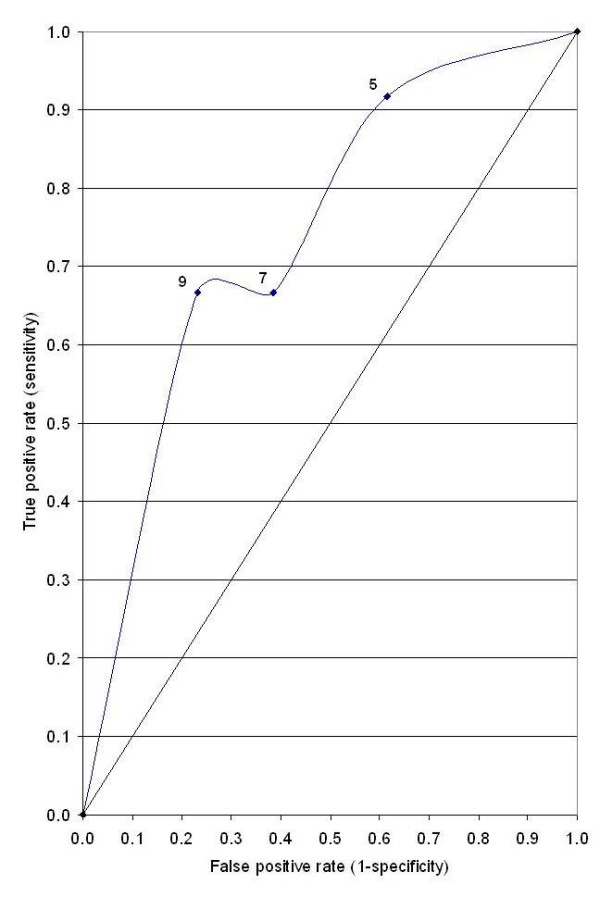
ROC curve for cumulative score.

The mean age of patients was 44.5 years, ranging from 30–65 years. The mean clinical diameter of the breast mass was 8.4 cm (range 6.7–14.6 cm). Forty four (88%) had T_4b _lesion at presentation. All patients showed the presence of hyper-vascular signals on pre-therapy color Doppler US. Complete response was seen in 24(48%) patients who could be correlated histologically. PI, RI and Vmax could not be measured in patients who had complete regression of the tumor. The grades of response in Doppler parameters following chemotherapy is shown in Table-1. Twenty two (44%) and 18(36%) of the 50 patients showed regression in RI and PI respectively while 4 (8%) and 8 (16%) patients respectively had increase in these parameters. Degree of changes in respect of RI, PI, Vmax varied according to grades of response. The mean measured values of PI, RI and Vmax at the time of presentation were 0.756 ± 0.246, 1.358 ± 0.546 and 0.396 ± 0.294 m/sec respectively. The cumulative Doppler scores were correlated with histopathological grades of response and found to be statistically significant (p < 0.05) (Table-2). Figures-1 and 2 show changes in color Doppler and Figure-3 shows a complete clinical response. The sensitivity and specificity at cumulative score >5 was 91.7% and 38.5% respectively. RI, PI & Vmax grade >3 and cumulative score >9 had the same values of diagnostic measures (Table-3). The ROC curves of RI, PI, Vmax at grade >3 (Figure-4) have the same area under the curve of 0.72. The ROC curve for the cumulative score of >5, >7 and >9 are shown in Figure-5. The area under the ROC curve of the cumulative score >9 is 0.72. These diagnostic tests are fair because the area under the curves lie between 0.7 to 0.8.

**Table 1:** Grades of response in Doppler parameters following chemotherapy

**Grades of response**	**RI** No. of patients (%)	**PI** No. of patients (%)	**Vmax** No. of patients (%)
1	22 (44%)*	18 (36%)**	2 (4%)
2	4 (8%)	6 (12%)	12 (24%)
3	0	2 (4%)	12 (24%)
4	24 (48%)	24 (48%)	24 (48%)

* 4 patients showed increase in RI, ** 8 showed increase in PI

**Table 2:** Histological response and Doppler cumulative score

	**Cumulative score of Doppler indices**			
		
**Histological response**	**3–5** No. of patients (%)	**6–9** No. of patients (%)	**10–12** No. of patients (%)	**Total**
2	4(8%)	2(4%)	2(4%)	8(16%)
3	6(12%)	8(16%)	4(8%)	18(36%)
4	2(4%)	6(12%)	16(32%)	24(48%)
**Total**	12(24%)	16(32%)	22(44%)	50(100%)

X^2 ^= 12.12, p < 0.05. Grade-1 response was not observed in any patient

**Table 3:** Comparison of Clinical, sonographic and Doppler response

**Modality**		**Sensitivity (%)**	**Specificity (%)**	**PPV (%)**	**NPV (%)**
Clinical		45.8	92.3	84.6	64.9
USG		33.3	88.5	72.7	59.0
Cummulative color Doppler score	>5	91.7	38.5	57.9	83.3
	>7	66.7	61.5	61.5	66.7
	>9	66.7	76.9	72.7	71.4
RI score	>1	91.7	53.8	64.7	87.5
	>2	66.7	69.2	66.7	69.2
	>3	66.7	76.9	72.7	71.4
PI score	>1	83.3	38.5	55.6	71.4
	>2	66.7	61.5	61.5	66.7
	>3	66.7	76.9	72.7	71.4
Vmax score	>1	100	7.7	50.0	100
	>2	91.7	38.5	57.9	83.3
	>3	66.7	76.5	72.7	71.4

## Discussion

Breast carcinoma significantly influences the women's health and is assuming greater importance in the developing countries due to the rising incidence, delay in presentation and dismal outcome. The advances in imaging modalities are not affordable to the financially compromised patients. Neoadjuvant chemotherapy offers a definite advantage by down staging the tumor, allowing less extensive surgery, controlling local and distant recurrence and providing a better quality of life. A steady decline in the mortality and increase in overall survival rates because of multimodality treatment is being contemplated in developing countries.

Rational use of neoadjuvant chemotherapy requires objective evaluation of chemotherapeutic response. Response assessment also helps in determining the early termination of ineffective regimens and change in the chemotherapeutic regimen [7]. The methods available are able to assess the response but predictability in an individual patient has not been documented. Various scoring systems have been advocated in breast cancer [5,8]. However, none of these are helpful in locally advanced carcinoma and can not predict chemotherapeutic response in patients undergoing neoadjuvant chemotherapy. This aspect has not been studied in available literature. An assessment of additional methods that identify changes in tumor physiology and vascularity were used for developing the proposed scoring system. Further, to assess the tumor behavior and response following therapy, a combination of two or more objective parameters must complement physical examination findings. The sensitivity of clinical examination alone for predicting pathological response was only 44.44% in one study [4]. Use of US as a response assessment tool has both objectivity and reproducibility. Vallone P *et al *[9] have concluded that ultrasound is the first step in assessing the efficacy of neoadjuvant chemotherapy in patients with locally advanced breast carcinoma.

Three dimensional tumor localization is better with sonography as it excludes the cutaneous edema. Dynamic magnetic resonance mammography and scintimammography have been used to assess the residual disease following chemotherapy [7,10,11]. However, these methods are not cost effective in developing countries. Color Doppler can be used to accurately measure the tumor vascularity changes following chemotherapy [12]. Contrary to dynamic MRI mammography, color Doppler is cost effective and easily accessible. The present study emphasizes its predictive value in response assessment against histopathology which is gold standard. The various parameters in a color Doppler study, besides the tumor size, are RI, PI, and Vmax. The RI has been found to be higher in malignant masses with sensitivity of up to 95.5% [13]. RI however depends on the presence of color flow in the tumor mass, and hence cannot be used as an independent predictor for response. The PI showed variable changes following chemotherapy. It was also dependent on the presence of flow signals in the tumor. Flow signals disappeared in patients with complete response. Corresponding changes in RI and PI have also been anticipated and confirmed in this study. All these color Doppler parameters put together can be used to assess independently the response of chemotherapy in breast cancer. Roubidoux *et al *[14], sonographically evaluated early stage breast cancer in 34 patients undergoing neoadjuvant chemotherapy and documented that the vascularity decreased following chemotherapy and was not specific for complete response. However, the present study observed that color Doppler is an independent predictor of chemotherapeutic response for locally advanced breast carcinoma. The gold standard for assessing response is histological changes in the form of histiocytosis, stromal fibrosis, calcification and lymphocytic infiltration. Since histopathological response is assessed in the postoperative specimen, color Doppler is an appropriate tool for pre-surgical assessment that correlates with the histopathological changes. We propose through this study a scoring system based on the different color Doppler parameters which would predict histopathological response in patients undergoing neoadjuvant chemotherapy. A score of more than 9 suggests a good response and less than 5 a poor response. A higher score showed statistically significant correlation with a histopathological response.

The objectives of the study were to evaluate the primary tumor mass only and formulate a scoring system based on primary tumor response assessed by color Doppler. The axillary lymph node status before or after induction chemotherapy has not been included in the present study. However, the study recommends further evaluation and correlation with response to chemotherapy of non-sentinel lymph nodes. Some independent parameters like ER, PR, erb-2/neu, VEGF etc. may predict response to induction chemotherapy and need correlation and could be another component of study. This being a pilot study with a view to suggest a scoring system based on color Doppler to assess response in the primary tumor, would act as a guide for further study.

The present study highlights the use of a cheaper modality to predict and monitor the response of advanced breast cancer to neoadjuvant chemotherapy. This is especially the need in the developing or third world countries where the cost of treatment is to be contained. By avoiding ineffective chemotherapy and reducing advanced surgery, the treatment will be more cost-effective in cases where the overall outlook is intrinsically bad. Color Doppler is an important and promising alternative to more expensive investigations. Multicenteric studies with longer follow-up periods will be required to establish this scoring system as a powerful tool in predicting the response and will have important therapeutic implications.

## Conclusion

Study of color Doppler vascularity changes in breast cancer directly correlates with histological response. Doppler scoring can be accurately used to objectively predict the response to chemotherapy in patients with locally advanced breast cancer. Higher score corresponded with a positive histological response. In the present study, forty four percent cases with complete response to chemotherapy had a score more than nine (p < 0.05). The sensitivity and specificity of the Doppler scoring, NPV and PPV in the study further supports the response assessment criteria. The scoring system suggested in the present study can predict the histopathological response in patients undergoing neoadjuvant chemotherapy.

## Competing interests

The author(s) declare that they have no competing interests.

## Authors' contributions

AK Conception, design and coordination of the study, SS acquisition of data and data analysis, SP Data analysis and interpretation and revising manuscript for its important intellectual content, RCS conducting the Doppler study and interpreted the Doppler findings, MAA Assisted in design and supervision of the study, TBS performed the statistical analysis and interpreted the results, RS was responsible for clinical study support and wrote the draft manuscript, SG carried out and interpreted the histological findings. All authors read and approved the final manuscript.
